# Children and adolescents hospitalized with chronic conditions: sleep patterns, resilience and quality of life*

**DOI:** 10.1590/1980-220X-REEUSP-2023-0363en

**Published:** 2024-04-29

**Authors:** Welker da Silva Xavier, Michelle Darezzo Rodrigues Nunes, Madalena Paulos Abreu, Lucila Castanheira Nascimento, Flávio Rebustini

**Affiliations:** 1 Universidade do Estado do Rio de Janeiro, Faculdade de Enfermagem, Rio de Janeiro, RJ, Brazil. Universidade do Estado do Rio de Janeiro Faculdade de Enfermagem Rio de Janeiro RJ Brazil; 2 Universidade de São Paulo, Escola de Enfermagem de Ribeirão Preto, Departamento de Enfermagem Materno-Infantil e Saúde Pública, Ribeirão Preto, SP, Brazil. Universidade de São Paulo Escola de Enfermagem de Ribeirão Preto Departamento de Enfermagem Materno-Infantil e Saúde Pública Ribeirão Preto SP Brazil; 3 Universidade de São Paulo, Escola de Artes, Ciência e Humanidades, Ribeirão Preto, SP, Brazil. Universidade de São Paulo Escola de Artes, Ciência e Humanidades Ribeirão Preto SP Brazil

**Keywords:** Child, Adolescent, Chronic Disease, Sleep Wake Disorders, Pediatric Nursing, Niño, Adolescente, Enfermedad Crónica, Trastornos del Sueño-Vigilia, Enfermería Pediátrica

## Abstract

**Objective:**

To evaluate the sleep pattern of children and adolescents with chronic conditions during hospitalization and correlate it with resilience, quality of life, clinical and sociodemographic data.

**Method:**

Quantitative, descriptive and cross-sectional study. Data collection took place between May 2022 and January 2023, with children and adolescents with chronic conditions from two hospitals in Rio de Janeiro. The instruments used were the Actigraph, Sandra Prince-Embury’s Resilience Scale for Children and Adolescents and the Pediatric Quality of Life Inventory. Data analysis involved descriptive statistics and correlation tests.

**Results:**

40 hospitalized children and adolescents between the ages of nine and 18 took part. The results showed compromised sleep, especially in terms of duration and time awake after sleep onset. Quality of life scores were low and resilience levels were classified as medium to high. Correlations were found between resilience and sleep. In addition, sleep was influenced by diagnosis and treatment.

**Conclusion:**

Children and adolescents hospitalized with chronic conditions experience significant sleep disturbances and have a low quality of life, but have satisfactory levels of resilience.

## RESUMEN

**Objetivo:**

Evaluar el patrón de sueño de niños y adolescentes con enfermedades crónicas durante la hospitalización y correlacionarlo con la resiliencia, la calidad de vida y los datos clínicos y sociodemográficos.

**Método:**

Estudio cuantitativo, descriptivo y transversal. La recolección de datos se realizó entre mayo de 2022 y enero de 2023, con niños y adolescentes con condiciones crónicas de dos hospitales de Río de Janeiro. Los instrumentos utilizados fueron el Actigraph, la Escala de Resiliencia para Niños y Adolescentes de Sandra Prince-Embury y el Inventario Pediátrico de Calidad de Vida. El análisis de los datos incluyó estadística descriptiva y pruebas de correlación.

**Resultados:**

Participaron 40 niños y adolescentes hospitalizados de entre 9 y 18 años. Los resultados mostraron un sueño comprometido, especialmente en términos de duración y tiempo despierto tras el inicio del sueño. Las puntuaciones de calidad de vida fueron bajas y los niveles de resiliencia se clasificaron entre medios y altos. Se hallaron correlaciones entre la resiliencia y el sueño. Además, el diagnóstico y el tratamiento influyeron en el sueño.

**Conclusión:**

Los niños y adolescentes hospitalizados con enfermedades crónicas experimentan alteraciones significativas del sueño y tienen una baja calidad de vida, pero presentan niveles satisfactorios de resiliencia.

## INTRODUCTION

Children and adolescents affected by chronic conditions are forced to learn to live with multiple, simultaneous and dynamic physical symptoms, such as pain, nausea, vomiting, loss of appetite and dyspnea, resulting from the diagnosis itself or from adverse effects and complications of treatment^(1)^. As a consequence, there is a need for recurrent hospitalization, which causes significant damage to social and school development, as well as psychosocial symptoms such as sadness, worry and changes in self-esteem^(1)^.

A chronic condition can affect the quality of life of children and adolescents in numerous ways, but one of the few discussed is sleep deprivation. Sleep disorders are a common complaint and affect around one in four children. The recognition and efficient management of these disorders are important to prevent damage to physical, cognitive, emotional, neurobehavioral and social development^(2)^.

Sleep is a basic physiological human need and essential for various aspects of human life and well-being. Assessing sleep is therefore an important aspect to consider during the overall physical examination of children and adolescents. When sleep is insufficient or of poor quality, numerous consequences can arise, not only related to physical aspects, but also psychological and social ones. In childhood, sleep becomes even more essential for physical and psychosocial growth and development^(3)^.

The prevalence of sleep disorders during childhood and adolescence is more frequent than one might think, ranging from 20 to 30% of the population of children and adolescents^(4)^. In children with chronic conditions, the main ones are worsening sleep quality and hygiene and difficulty falling asleep. When they are hospitalized, they tend to sleep less, due to the characteristics of their diagnosis, the structure of the hospitalization areas and the care provided by the healthcare team^(5)^.

Even after discharge from hospital, it is possible to observe changes in the sleep patterns of these children and adolescents with cancer. A study using ActiGraph at home showed that they had a sleep duration of approximately six hours and that the shorter this time, the more prone they were to fatigue problems. Sleep was also found to be interrupted for around one hour for children and two hours for adolescents^(6)^.

The effects of fragmented sleep can also be quite significant in the recovery of children and adolescents, as sleep deficit can compromise the immune system, the body’s ability to respond and pain tolerance^(7)^. Other consequences include loss of attention, irritability, impulsiveness, decreased tolerance to frustration, learning difficulties, vulnerability to accidents, mood dysfunctions and the development of diseases in adulthood, such as hypertension, diabetes and obesity^(3)^.

Therefore, there is objective and subjective evidence of frequent sleep disorders in various chronic health conditions, which reiterates the importance of considering this aspect in the care of children and adolescents. The literature highlights reports of poorer sleep quality during hospitalization, susceptibility to pain and discomfort, lighting and noise in the room, distance from home and anxiety about clinical procedures^(8)^. In addition, there is evidence that these problems can remain even after discharge^(5)^.

With this in mind, the general objective of this study is to evaluate the sleep patterns of children and adolescents with chronic conditions during hospitalization. The specific objectives are to explore factors potentially capable of affecting sleep, such as age, gender, skin color, diagnosis, treatment, length of stay, other chronic conditions and reason for hospitalization, and to correlate sleep pattern, resilience and quality of life in children and adolescents with chronic conditions.

## METHOD

### Study Design

This is a quantitative, descriptive and exploratory cross-sectional study.

### Study Site

The study sites were two referral hospitals in the city of Rio de Janeiro (RJ) for the treatment of children and adolescents with chronic health conditions. The pediatric ward, infectious disease ward and adolescent ward were included.

### Population and Selection Criteria

The non-probabilistic sample consisted of children and adolescents with chronic conditions, aged between nine and 18 incomplete years, admitted to the two public reference hospitals selected for the study. The choice of age group is justified since the sleep data was correlated with others that have analyzed the resilience of children and adolescents with chronic conditions and the scale used for this analysis is aimed exclusively at this age group.

The inclusion criteria were children and adolescents diagnosed with a chronic condition, aged between nine and 18 and of both sexes. Exclusion criteria were those who were in the immediate post-operative period, hospitalized in intensive care units and unable to answer the questionnaire. Specifically for the collection of sleep data, only children and adolescents with a minimum stay of three full days in the wards were included because, according to the recommendations for using the ActiGraph, the device must be used for at least three consecutive 24-hour days for the data to be reliable^(9)^. Children and adolescents with malformations that made it impossible to fit the ActiGraph device for sleep collection were excluded.

### Data Collection

Data collection was done between May 2022 and January 2023. To characterize the participants, a form was used to summarize clinical data (diagnosis, time since diagnosis, diagnosis of other chronic conditions, treatment carried out and reason for hospitalization) and sociodemographic data (age, gender, skin color and schooling).

The instrument used to collect data on the participants’ sleep was the ActiGraph wristband, which remained on the wrist of the children and adolescents for a period of at least three consecutive and complete days. The accumulated data was initially stored on the device itself (Mini Motionlogger ActiGraph Ambulatory Monitoring, Inc) and, after removing the device, transferred via an interface to a notebook using specific software, WatchWare, version 1.99.34.1. With other software, Action W, version 2.7, the data was processed and expressed in the form of actograms. The variables used in this study were: sleep duration, wake after sleep onset (WASO), sleep efficiency and sleep percent. It is worth noting that WASO and sleep duration are calculated in minutes, while sleep percentage and sleep efficiency are calculated in percentages^(10)^.

Actigraphy is a valid and reliable tool for assessing sleep, with high correlations between the activities measured, both at the wrist and at the waist: nocturnal activity, with r = 0.91 and p < 0.001; sleep duration, with r = 0.78 and p < 0.001; sleep latency, with r = 0.78 and p < 0.001; sleep percentage, with r = 0.89 and p < 0.001, and sleep efficiency, with r = 0.91 and p < 0.001^(11)^. In addition, sleep estimation using ActiGraph shows that the correlation with polysomnography is around 90%, which is considered the gold standard for detecting sleep disorders^(12)^.

In order to assess resilience, we used Sandra Prince-Embury’s Resilience Scale for Children and Adolescents^(13)^, a version of which was translated and validated into Portuguese in 2008 with children and adolescents aged between nine and 18^(14)^. It consists of three subscales: control, relationship skills and emotional reactivity. Each contains between 20 and 24 items which, in total, assess ten factors. For each item, there are five alternatives to choose from, with Likert answers. It is filled in using the child’s and adolescent’s self-report and classifies each of the sub-scales as: low (≤ 40), below average (41 to 45), average (46 to 55), above average (56 to 59) and high (≥ 60). In the sub-scales of control and ability to relate, scores classified as average, above average and high indicate an individual’s ability to experience these areas satisfactorily. On the other hand, in the emotional reactivity subscale, a score above average or high indicates potential for vulnerability^(13,14).^

The psychometric parameters of the validated Brazilian version were satisfactory in relation to the Prince-Embury version in all three scales, indicating good internal consistency of the instrument. Cronbach’s alpha coefficient was 0.83 for the control subscale, 0.80 for the relationship capacity subscale, 0.87 for the emotional reactivity subscale and 0.93 for the total resilience scale^(14)^. In the original study, the author found values of 0.95, 0.95 and 0.94, respectively^(13)^.

To assess quality of life, we used the Pediatric Quality of Life Inventory generic module 4.0 - PedsQL™. This is a modular instrument designed to measure Health-Related Quality of Life (HRQoL) in children and adolescents aged between two and 18 years, divided by age group^(15)^. The self-report versions for children (aged eight to 12) and adolescents (aged 13 to 18) were used. It has four subscales: physical functioning, emotional functioning, social functioning and school functioning. Each scale uses a five-point Likert-type scale to ask the child/adolescent or caregiver how much of a problem a particular item has been over the previous week. The raw scores are transformed into scales from zero to one hundred, where 1 = 100, 2 = 75, 3 = 50, 4 = 25, 5 = 0. Higher scores indicate better HRQoL^(15)^. The instrument was created by Varni, Seid and Rode (1999) and cross-culturally validated in several countries, including translation and validation into Brazilian Portuguese in 2008^(16)^.

Regarding ethical authorial aspects, prior contact was made with the authors of the scales for the use of the instruments, and permission was granted.

In order to ensure the internal and external validity of the study and guarantee reliable results without distortions from potential sources of bias, the research team was trained before starting data collection to standardize the application of the data collection instruments, reducing the possibility of errors. With regard to sample selection, clear eligibility criteria were established for the participants. In addition, during data collection, an attempt was made to ensure that the sample was representative, using a non-probabilistic approach for convenience, in order to guarantee the inclusion of all relevant strata of the population.

### Data Analysis and Processing

For the statistical analyses, the scores obtained from the HRQoL and resilience scales were initially calculated and the data extracted from ActiGraph. A database was created in an Excel spreadsheet (Microsoft Office) to code the variables, with double entry carried out by different researchers. The data was then exported to the JASP statistical software, version 0.17.1, to carry out the appropriate statistical tests.

To characterize the sample, the continuous dependent variables of the participants’ sociodemographic data were described in mean and standard deviation, and the nominal dependent variables in absolute (n) and relative (%) frequency distributions. The ordinal dependent variables (sleep scores, HRQoL and resilience) were reported by analyzing measures of central tendency (mean and median -Md-) and dispersion (standard deviation, interquartile range (IQR), minimum, maximum and range).

Since the answers to the questionnaires on the Likert scale are categorical/ordinal, the mean and standard deviation values were used for reference purposes only, and the median measures for describing the results. The analysis of variance applied to the study was therefore non-parametric.

In the inferential analysis of the associations between the sleep, HRQoL and resilience scores and the participants’ characteristics, statistical significance tests were carried out between the distributions of responses from different subgroups. Non-parametric Mann-Whitney tests were used for binary variables, and Kruskal-Wallis, with post-hoc Dunn’s test and Bonferroni correction to control for type I error, for polytomous variables.

Finally, to describe the associations between sleep, HRQoL and resilience, Kendall’s Tau correlation was used, given the small number of data, and considering that a coefficient is significantly non-zero if the p-value of the correlation test is <0.05. Correlation values between 0.10 and 0.29 indicate no or little correlation; values between 0.30 and 0.49 show that there is a medium correlation and those between 0.50 and 1 can be interpreted as a high correlation^(17)^.

### Ethical Aspects

Initially, the proposal was submitted to the Ethics Committee for Research with Human Beings (CEP) of the proposing institution, which received approval and was registered under opinion 5.299.621. The next step was to obtain the agreement of the co-participating institution, which also responded favorably to the research, under opinion 5.376.441. The entire development of this research was based on Resolution 466/12 of the National Health Council.

Each participant in the study, child or adolescent and guardian, voluntarily expressed their desire to take part in the study or not. The process of obtaining consent ended with parents or guardians signing an informed consent form and minors signing an informed consent form.

## RESULTS

Forty children and adolescents with chronic conditions, aged between nine and 18, hospitalized in one of the selected institutions, took part in the study. All were asked to complete the resilience and HRQoL scales, but only 26 completed the sleep assessment data.

With regard to the sociodemographic characterization of the participants, the average age was 13.7 ± 2.6 years. Most of them were adolescents aged between 13 and 18 (n = 30; 75%), and the other 25% were children aged between nine and 12 (n = 10); 67.5% were female (n = 27) and 32.5% were male (n = 13); 60% declared themselves to be brown (n = 24), 22.5% white (n = 9) and 17.5% black (n = 7); finally, 77.5% were in elementary school (n = 31) and 22.5% in secondary school (n = 9).

Regarding the classification of the diagnosis, 12 (30%) had some genetic condition, 12 (30%) some autoimmune condition and the rest (n = 16; 40%) were grouped as other chronic conditions.

At the time of data collection, the majority of participants (n = 15; 37.5%) had been diagnosed for 10 years or more, 22.5% between one and three years (n = 9), 20% between four and nine years (n = 8), 15% between one and 12 months (n = 6) and only 5% were unable to answer (n = 2). The majority did not have a diagnosis of another chronic condition (n = 33; 82.5%) and only 17.5% had another associated condition (n = 7). Finally, the main reason for hospitalization was acute or clinical (n = 24; 60%), followed by complications of the chronic condition itself (n = 8; 20%), drug treatment (n = 6; 15%) and other causes (n = 2; 5%). With regard to treatment, 50% of the participants were exclusively medicated (n = 20) and the other 50% combined multiple treatment modalities (n = 20).

Descriptive data on the participants’ HRQoL is shown in Table 1. Overall, the Md of the total HRQoL scores and their dimensions was low, indicating problems often or almost always. The Md of the total HRQoL was 60.89 (IQR = 22.83). Furthermore, of all the dimensions, the one with the highest score was “social functioning” (Md = 77.50; IQR = 26.25), and the ones with the lowest scores were “emotional functioning” (Md = 50.00; IQR = 25.00) and “school functioning” (Md = 50.00; IQR = 31.25) (Table 1).

**Table 1:** Quality of life, resilience and sleep variables of children and adolescents participating in the study – Rio de Janeiro, RJ, Brazil, 2023.

Subscales	N	Md	IQR	Amp	Min	Max	Mean ± SD
HRQOL							
Physical functioning	40	64,06	38,28	93,75	3,12	96,87	60,62 ± 22,86
Emotional functioning	40	50,00	25,00	85,00	15,00	100,00	52,00 ± 20,62
Social functioning	40	77,50	26,25	70,00	30,00	100,00	74,75 ± 18,50
School functioning	40	50,00	31,25	80,00	10,00	90,00	50,05 ± 19,38
Total generic HRQoL	40	60,86	22,83	59,78	25,00	84,78	59,36 ± 15,18
Resilience							
Control	40	52,00	20,50	54,00	22	76	50,44 ± 13,36
Relationship capacity	40	66,00	23,50	61,00	35	96	68,35 ± 15,67
Emotional reactivity	40	26,50	19,75	45,00	06	51	27,20 ± 13,54
Sleep variables							
WASO	26	39,62	31,65	106,05	10,20	116,25	42,47 ± 26,14
Sleep duration	26	221,27	109,35	346,03	69,22	415,25	216,42 ± 87,59
Sleep, %	26	70,16	16,35	51,71	41,28	92,99	70,05 ± 14,91
Sleep efficiency	26	89,43	7,90	20,30	75,68	95,98	87,76 ± 5,89

Md: Median; IQR: Interquartile Range; Amp: Range; Min: Minimum; Max: Maximum; SD: Standard Deviation; HRQoL: Health-Related Quality of Life; WASO: Wake After Sleep Onset.

With regard to resilience, the descriptive results are shown in Table 1. The median scores for the resilience subscales were 52 (IQR = 20.50) for control, 66 (IQR = 23.50) for relationship capacity and 26.50 (IQR = 19.75) for emotional reactivity. It should be noted that, with these results, the participants could be classified as having medium resilience for the control subscale and high resilience for the relationship capacity subscale, which represents a satisfactory ability to deal with life experiences. In the emotional reactivity subscale, resilience was rated as low, which did not indicate vulnerability for these individuals (Table 1).

Since not all the children and adolescents selected for the study remained in hospital for three full days, sleep data was collected on part of the participants (n = 26; 65%). The descriptive results of the participants’ sleep patterns are shown in Table 1.

It was noted that, after starting to sleep, the children and adolescents remained awake for between 10.2 and 116.3 minutes, with a Md of 39.62 minutes (IQR = 31.65) awake. They slept a mean of 221.27 minutes (IQR = 109.35), which was equivalent to approximately 3.7 hours, with a minimum of 69.2 minutes (1.1 hours) and a maximum of 415.3 minutes (6.9 hours) between sleep and wake intervals. This result shows the compromised sleep pattern of the study participants.

With regard to sleep percentage, the Md was 70.16% (IQR = 16.35), with a minimum of 41.3% and a maximum of 93%. Sleep efficiency reached an Md of 89.43% (IQR = 7.90), ranging from 75.3 to 96%.

The inferential analysis of the associations between sleep scores and the participants’ clinical and sociodemographic characteristics showed that the following factors had no influence on sleep: age, gender, diagnosis of other chronic conditions, length of diagnosis and reason for hospitalization. However, the Kruskal-Wallis analysis of variance indicated a violation of the null hypothesis in relation to diagnosis, specifically for the sleep efficiency variable (H = 6.22; p = 0.04) (Table 2).

**Table 2:** Results of the Kruskal-Wallis test for sleep variables in children and adolescents participating in the study, according to diagnosis – Rio de Janeiro, RJ, Brazil, 2023.

Sleep variables	Factor	Statistics (H)	Df	P-value
WASO	Diagnostic	3,42	2	0,18
Sleep duration	Diagnostic	1,94	2	0,38
Sleep, %	Diagnostic	3,73	2	0,15
Sleep efficiency	Diagnostic	6,22	2	0,04*

*p < 0,05. Df: degrees of freedom; WASO: *Wake After Sleep Onset.*

When the Bonferroni correction was applied, the violation of the null hypothesis was maintained, specifically in the comparison between genetic and autoimmune conditions (Pbonf = 0.04) (Table 3).

**Table 3:** Post-hoc Dunn’s test for comparisons between diagnoses – Rio de Janeiro RJ, Brazil, 2023.

Sleep variables	Comparisons	Z	Wi	Wj	Pbonf
WASO	Genetics – autoimmune	1,75	16,43	9,50	0,24
	Genetics – other	0,51	16,43	14,54	1,00
	Autoimmune – other	–1,42	9,50	14,54	0,47
Sleep duration	Genetics – autoimmune	0,43	12,71	11,00	1,00
	Genetics – other	–0,84	12,71	15,82	1,00
	Autoimmune – other	–1,36	11,00	15,82	0,53
Sleep, %	Genetics – autoimmune	0,37	11,86	10,37	1,00
	Genetics – other	–1,34	11,86	16,82	0,54
	Autoimmune – other	–1,81	10,37	16,82	0,21
Sleep efficiency	Genetics – autoimmune	–2,47	8,71	18,50	0,04*
	Genetics – other	–1,13	8,71	12,91	0,77
	Autoimmune – other	1,57	18,50	12,91	0,35

* p < 0,05. Wi and Wj: sums of ranks in treatments i and j, respectively; Pbonf: Bonferroni’s p; WASO: Wake After Sleep Onset.

Descriptive analyses showed that participants diagnosed with genetic conditions (Md = 83.55; IQR = 2.26) had lower sleep efficiency than those with autoimmune conditions (Md = 91.90; IQR = 3.61) (Table 4).

**Table 4:** Sleep variables of children and adolescents participating in the study, according to diagnosis and treatment – Rio de Janeiro, RJ, Brazil, 2023.

Sleep variables	Groups	N	Md	IQR	Amp	Min	Max	Mean ± SD
WASO	Genetics	7	44,60	29,47	79,33	21,50	100,83	51,91 ± 27,31
	Autoimmune	8	28,66	17,18	60,80	10,20	71,00	29,52 ± 19,33
	Other	11	46,00	28,04	104,12	12,13	116,25	45,87 ± 28,10
Sleep duration	Genetics	7	199,57	82,36	207,07	88,50	295,57	200,64 ± 71,83
	Autoimmune	8	185,73	105,47	293,89	84,44	378,33	195,37 ± 96,54
	Other	11	246,44	68,72	346,03	69,22	415,25	241,78 ± 91,25
Sleep, %	Genetics	7	70,01	15,21	41,02	41,28	82,30	66,54 ± 14,89
	Autoimmune	8	63,92	17,97	48,30	42,70	91,00	64,46 ± 16,53
	Other	11	78,10	14,15	42,98	50,01	92,99	76,35 ± 12,42
Sleep efficiency	Genetics	7	83,55	2,26	18,81	75,68	94,49	84,22 ± 5,57
	Autoimmune	8	91,90	3,61	20,06	75,92	95,98	90,92 ± 6,42
	Other	11	88,84	4,06	15,02	78,10	93,12	87,71 ± 4,76
WASO	Medication	15	46,00	40,67	105,00	11,25	116,25	50,72 ± 29,96
	Multiple	11	30,33	17,12	44,940	10,20	55,14	31,21 ± 14,48
Sleep duration	Medication	15	199,57	106,41	330,81	84,44	415,25	216,23 ± 89,14
	Multiple	11	230,71	96,49	309,11	69,22	378,3	216,69 ± 89,76
Sleep, %	Medication	15	70,31	15,38	51,60	41,28	92,88	70,08 ± 15,09
	Multiple	11	70,02	15,65	50,23	42,76	92,99	70,01 ± 15,40
Sleep efficiency	Medication	15	84,37	7,41	20,21	75,68	95,89	85,02 ± 6,29
	Multiple	11	91,00	1,95	8,08	87,90	95,98	91,50 ± 2,17

Md: Median; IQR: Interquartile Range; Amp: Range; Min: Minimum; Max: Maximum; SD: Standard Deviation; WASO: Wake After Sleep Onset.

Furthermore, when sleep was assessed according to treatment, the results of the Mann-Whitney test also indicated a violation of the null hypothesis, specifically for the sleep efficiency variable (W = 31.00; p = 0.01) (Table 5).

**Table 5:** Results of the Mann-Whitney test for sleep variables in children and adolescents participating in the study, according to treatment – Rio de Janeiro, RJ, Brazil, 2023.

Sleep variables	Statistic (W)	Rank-biserial	VS-MPR	P-value
WASO	116,00	0,41	1,73	0,09
Sleep duration	80,00	–0,03	1,00	0,92
Sleep, %	85,00	0,03	1,00	0,92
Sleep efficiency	31,00	–0,62	11,31	0,01*

* p < 0,05. VS-MPR: Vovk-Sellke maximum p-ratio; WASO: Wake After Sleep Onset.

It can be seen that the children and adolescents treated exclusively with medication had worse sleep efficiency (Md = 84.37; IQR = 7.41) when compared to those who added other treatment modalities (Md = 91.00; IQR = 1.95) (Table 4).

With regard to the correlation tests, no significant correlations were found between the total HRQoL score, as well as its physical, emotional, social and school functioning dimensions, with any of the four sleep variables used (waking after sleep onset, sleep duration, sleep percentage and sleep efficiency).

A mean positive correlation was found between the emotional reactivity subscale of resilience and the variable waking up after sleep onset (r = 0.39; p = 0.005), as well as a mean negative correlation with sleep efficiency (r = –0.31; p = 0.03), i.e. participants with a lower emotional reactivity score and, consequently, better resilience, woke up less after sleep onset and had better sleep efficiency. No correlations were found between the other resilience subscales and other sleep variables.

## DISCUSSION

According to the Portuguese Sleep Society, it is recommended that children and adolescents between the ages of six and 13, have sleep time between nine and 11 hours; between the ages of 14 and 17, around eight to 10 hours; and, from the age of 18, between seven and nine hours^(18)^. It may therefore be seen that the individuals taking part in the study had a total sleep time well below the recommended level. Adequate, quality sleep is defined by: at least 85% efficiency; being able to fall asleep in 30 minutes or less; waking up no more than once during the night; and staying awake for less than 20 minutes after sleep begins^(19)^. As a result, the time that the children in the study remained awake after the initial phase of sleep was also compromised.

Concurring with the above findings, a new multicenter study with Brazil, Portugal and the United States, using the ActiGraph, showed that during hospitalization, children and adolescents with cancer have decreased sleep percentage and efficiency values (approximately 59% and 79%, respectively), regardless of pain symptoms^(20)^. There are numerous risks related to sleep deprivation, such as the presence of short- and long-term physical and psychological disorders. When total sleep time is still less than four hours, the decrease in cognitive performance of individuals is potentiated^(3)^.

In the present study, no violations of the null hypothesis were found for the sleep assessment variables according to age and gender. Similar results were found in a study of children and adolescents aged between 10 and 18 with type I diabetes mellitus, for whom age and gender were not predictors of a greater chance of daytime sleepiness^(21)^. There are also compatible results with children and adolescents with cancer, and age was also not a predictor of shorter sleep duration^(6)^.

Regarding the other sociodemographic data, such as place of study, skin color, schooling, gender, schooling and marital status of the guardian, origin and family income, no violations of null hypotheses were found for the sleep variables. However, another study corroborates this result. In a study of children and adolescents aged between 13 and 18 with chronic gastritis, it was found that the quality of sleep was significantly worse in the group with an income lower than their actual financial expenditure^(22).^

This study found a violation of the null hypothesis for sleep efficiency according to the diagnosis of the participants (Pbonf = 0.04), showing that those with genetic chronic conditions had lower efficiency compared to those with autoimmune conditions. Treatment also had a negative influence on the sleep efficiency variable, and the group of participants undergoing exclusively drug treatment had lower values when compared to the multiple treatment group.

Aligned with these results, a study of 30 children with cystic fibrosis, polysomnography analysis showed a reduction in sleep efficiency (66.5%), an 8.7% awakening rate, a 2.6% Md breathing disorder rate and obstructive sleep apnea syndrome in 30% of the participants^(23)^.

Neither time of diagnosis, reason for hospitalization and diagnosis of other chronic conditions did significantly affect sleep variables, but other studies have found opposite results. In children and adolescents with type I diabetes mellitus, there was a higher prevalence of daytime sleepiness among patients with a diagnosis of less than 3 years when compared to those with a diagnosis of 3 years or more (57.1% versus 36.2%; p = 0.03). However, insulin treatment had no influence on daytime sleepiness (p = 0.80)^(21)^.

No significant correlations were found between the total HRQoL score and its dimensions with any of the four sleep variables used. On the other hand, other studies have found associations, showing the influence between HRQoL and sleep. A study of adolescents aged between 10 and 16 with type I diabetes mellitus showed a significant correlation (p = 0.02) between sleep quality, as measured by the PSQI, and the physical domain of the World Health Organization Quality of Life bref questionnaire (WHOQOL-bref). In these individuals, the worse the quality of sleep, the worse the quality of life in the physical domain^(24).^

Recent studies have also shown the influence of the Covid-19 pandemic on the quality of life and sleep of children and adolescents with chronic conditions. In subjects with nephrotic syndrome aged between 6 months and 18 years, overall HRQoL decreased (p = 0.04), while sleep disruption increased and energy decreased (p = 0.01) during the pandemic. In addition, an increase in the child’s age was also a predictor of greater sleep disruption and a decrease in overall quality of life^(25)^.

With regard to resilience and sleep, this study found that the higher the resilience emotional reactivity score, the longer the time spent awake after starting sleep, and the lower the sleep efficiency. Coping strategies are also important indicators of resilience and emotional reactivity performance, which consists of the ability to maintain balance after disturbances or strong arousals. These strategies represent behavioral and cognitive modifications through which individuals alter their environment and emotions to deal with challenging and stressful situations^(26)^.

As the chronic condition arises, these positive coping strategies are also necessary to re-establish a balance between the physical complications, signs and symptoms and meeting their needs, as well as social limitations, such as school avoidance. As a repercussion of this isolation and limitations, various symptoms can be triggered or exacerbated, including anxiety, depression, insomnia and excessive sleepiness^(1)^.

Although the relevance of the subject has been proven, there is a lack of research on children and adolescents with chronic conditions, which highlights the importance of this study. However, some limitations were found, which constitute possibilities for further research. It is known that the sample size is small, a fact justified by the need for prolonged hospitalization of the participants selected for the study, and this could significantly interfere with the results.

Studies similar to the present^(6,11,20)^ are a challenge for researchers, given the scarcity of literature relating sleep and the variables used here. This reiterates the fragility and gap in studies in the area, and is an important topic to be addressed in future studies that overcome the limitations found.

It is known that children and adolescents with chronic conditions are more susceptible to developing sleep disorders, such as decreased sleep quality and hygiene, nocturnal awakenings and difficulty initiating sleep. In hospitalization situations, these problems are exacerbated by the characteristics of the condition itself, treatment, multiple symptoms, noise and lighting and the care provided by the nursing staff^(27)^. In this context, various strategies can be used to provide safe, quality and holistic nursing care, such as the use of therapeutic toys, distraction techniques during procedures, guided imagination, relaxation with deep breathing and drawing, among others^(28)^.

## CONCLUSION

The results show that sleep in this population is significantly impaired during hospitalization as postulated in the purpose of this study. In addition, poorer sleep efficiency was found in participants diagnosed with genetic conditions and taking only medication. With regard to resilience, the better the emotional reactivity, the shorter the time participants spent awake after the initial sleep phase and the better their sleep efficiency.

It is well-known that the consequences of sleep deprivation also need to be assessed by health professionals, as these individuals become more susceptible to delayed growth and development, the development of other chronic conditions, learning difficulties, behavioral changes and cognitive impairment.

Thus, the first implication for nursing refers to the inclusion of sleep pattern assessment, using valid and reliable instruments when planning their activities. To do this, nurses need to be aware of the structure and duration of sleep for each age group, as well as the instruments available for this purpose.

Once the sleep disorders in children and adolescents with chronic conditions during hospitalization are evident, it becomes urgent to implement strategies to minimize them. This can be done, for example, by maintaining a cozy environment and practicing massage and music therapy, which can be included in the nursing process to ensure quality sleep in this population.

Therefore, it is important to evaluate sleep patterns early on, so that children and adolescents can have a better quality of life and resilience during the period of hospitalization and treatment, by properly managing this symptom. However, further studies, with a larger number of participants and longer data collection times, are necessary and should be encouraged in order to delve deeper into the subject in the child and adolescent age group.

## References

[1] 1. Silva VE, Nunes MDR, Macedo IF, Possi JCS, Silva-Rodrigues FM, Pacheco STA. Convivendo com múltiplos sintomas: a experiência de crianças e adolescentes com condição crônica. Rev Enf UERJ. 2020;28:e47474. doi: http://dx.doi.org/10.12957/reuerj.2020.47474.

[2] 2. Fernandes AER, Santos CF. Manejo dos principais distúrbios do sono em pediatria. Rev Med Minas Gerais. 2019 [cited 2023 Nov 17];29:S62–7. Available from: https://rmmg.org/artigo/detalhes/2626.

[3] 3. Vecchi CR. Causes and effects of lack of sleep in hospitalized children. Arch Argent Pediatr. 2020;118(2):e143–7. doi: http://dx.doi.org/10.5546/aap.2020.eng.e143. PubMed PMID: 32199053. 10.5546/aap.2020.eng.e143 32199053

[4] 4. Honaker SM, Meltzer LJ. Sleep in pediatric primary care: a review of the literature. Sleep Med Rev. 2016;25:31–9. doi: http://dx.doi.org/10.1016/j.smrv.2015.01.004. PubMed PMID: 26163054. 10.1016/j.smrv.2015.01.004 26163054

[5] 5. Setoyama A, Ikeda M, Kamibeppu K. Objective assessment of sleep status and its correlates in hospitalized children with cancer: exploratory study. Pediatr Int. 2016;58(9):842–9. doi: http://dx.doi.org/10.1111/ped.12927. PubMed PMID: 26767328. 10.1111/ped.12927 26767328

[6] 6. Nunes MDR, Jacob E, Adlard K, Secola R, Nascimento L. Fatigue and sleep experiences at home in children and adolescents with cancer. Oncol Nurs Forum. 2015;42(5):498–506. doi: http://dx.doi.org/10.1188/15.ONF.498-506. PubMed PMID: 26302278. 10.1188/15.ONF.498-506 26302278

[7] 7. Santana TP, Muniz JA, Lopes MC, Lúcio MF, dos Santos ABB, Ahumada DAR, et al. Sleep and immunity: role of the immune system, sleep disorders and treatment. Braz J Dev. 2021;7(6):55769–84. doi: http://dx.doi.org/10.34117/bjdv7n6-131.

[8] 8. Anggerainy SW, Wanda D, Nurhaeni N. Music therapy and story telling: nursing interventions to improve sleep in hospitalized children. Compr Child Adolesc Nurs. 2019;42(sup1):82–9. doi: http://dx.doi.org/10.1080/24694193.2019.1578299. PubMed PMID: 31192722. 10.1080/24694193.2019.1578299 31192722

[9] 9. Littner M, Kushida CA, Anderson WM, Bailey D, Berry RB, Davila DG, et al.; Standards of Practice Committee of the American Academy of Sleep Medicine. Practice parameters for the role of ActiGraphy in the study of sleep and circadian rhythms: an update for 2002. Sleep. 2003;26(3):337–41. doi: http://dx.doi.org/10.1093/sleep/26.3.337. PubMed PMID: 12749556. 10.1093/sleep/26.3.337 12749556

[10] 10. Sadeh A, Raviv A, Gruber R. Sleep patterns and sleep disruptions in school-age children. Dev Psychol. 2000;36(3):291–301. doi: http://dx.doi.org/10.1037/0012-1649.36.3.291. PubMed PMID: 10830974. 10.1037//0012-1649.36.3.291 10830974

[11] 11. Paavonen EJ, Fjällberg M, Steenari MR, Aronen ET. Actigraph placement and sleep estimation in children. Sleep. 2002;25(2):235–7. doi: http://dx.doi.org/10.1093/sleep/25.2.235. PubMed PMID: 11902433. 10.1093/sleep/25.2.235 11902433

[12] 12. Souza L, Benedito-Silva AA, Pires MLN, Poyares D, Tufik S, Calil HM. Further validation of actigraphy for sleep studies. Sleep. 2003;26(1):81–5. doi: http://dx.doi.org/10.1093/sleep/26.1.81. PubMed PMID: 12627737. 10.1093/sleep/26.1.81 12627737

[13] 13. Prince-Embury S. Resiliency scale for adolescents: a profile of personal strengths. Harcourt Assessment. 2007;22(2):255–61. doi: http://dx.doi.org/10.1177/0829573507305520.

[14] 14. Barbosa RJ. Tradução e validação da escala de resiliência para crianças e adolescentes de Sandra Prince-Embury [dissertação]. São Paulo: Pontifícia Universidade Católica de São Paulo; 2009 [cited 2023 Sep 12]. Available from: http://tede2.pucsp.br/tede/handle/handle/15844

[15] 15. Varni JW, Seid M, Kurtin PS. PedsQL 4.0: reliability and validity of the Pediatric Quality of Life Inventory version 4.0 generic core scales in healthy and patient populations. Med Care. 2001;39(8):800–12. doi: http://dx.doi.org/10.1097/00005650-200108000-00006. PubMed PMID: 11468499. 10.1097/00005650-200108000-00006 11468499

[16] 16. Klatchoian DA, Len CA, Terreri MTRA, Silva M, Itamoto C, Ciconelli RM, et al. Qualidade de vida de crianças e adolescentes de São Paulo: confiabilidade e validade da versão brasileira do questionário genérico Pediatric Quality of Life InventoryTM versão 4.0. J Pediatr. 2008;84(4):308–15. doi: http://dx.doi.org/10.1590/S0021-75572008000400005. 10.2223/JPED.1788 18679557

[17] 17. Cohen J. Statistical power analysis for the behavioral sciences. 2nd ed. Hillsdale: L. Erlbaum Associates; 1988.

[18] 18. Sociedade Portuguesa do Sono. Recomendações SPS e SPP: prática da sesta da criança nas creches e infantários, públicos ou privados [Internet]. Lisboa: SPP; 2017 [cited 2023 Aug 12]. Available from: https://www.spp.pt/UserFiles/file/Noticias_2017/VERSAO%20PROFISSIONAIS%20DE%20SAUDE_RECOMENDACOES%20SPS-SPP%20SESTA%20NA%20CRIANCA.pdf.

[19] 19. Angelhoff C, Sjølie H, Mörelius E, Løyland B. “Like Walking in a Fog”: Parents’ perceptions of sleep and consequences of sleep loss when staying overnight with their child in hospital. J Sleep Res. 2019;29(2):e12945. doi: http://dx.doi.org/10.1111/jsr.12945. PubMed PMID: 31724227. 10.1111/jsr.12945 31724227

[20] 20. Nunes MDR, Nascimento LC, Fernandes AM, Batalha L, Campos C, Gonçalves A, et al. Pain, sleep patterns and health-related quality of life in paediatric patients with cancer. Eur J Cancer Care. 2020;28(4):e13029. doi: http://dx.doi.org/10.1111/ecc.13029. PubMed PMID: 30828888. 10.1111/ecc.13029 30828888

[21] 21. Silva RA, Ganen ADP, Fernandes VFT, Evangelista NMA, Figueiredo CC, Pacheco LA, et al. Avaliação das características do sono em crianças e adolescentes portadores de diabetes melito tipo 1. Rev Paul Pediatr. 2021;40:e2020407. doi: http://dx.doi.org/10.1590/1984-0462/2022/40/2020407. PubMed PMID: 34614139. 10.1590/1984-0462/2022/40/2020407 PMC8543827 34614139

[22] 22. Appak YÇ, Özyurt G, Karakoyun M, Baran M. Effects of chronic gastritis on sleep and quality of life in adolescents. J Pediatr Res. 2019;6(4):1. doi: http://dx.doi.org/10.4274/jpr.galenos.2019.60134.

[23] 23. Lugao RS, Barbosa RRB, Coelho PF, Liberato FMG, Vidal PR, Carvalho RBCO, et al. Associação de distúrbios do sono com a variabilidade da frequência cardíaca em crianças e adolescentes com fibrose cística. Rev Paul Pediatr. 2021;40:e2020295. doi: http://dx.doi.org/10.1590/1984-0462/2022/40/2020295. PubMed PMID: 34495277.

[24] 24. Cordeiro GR, Smolarek AC, Smouter L, Rebesco DB, Ferreira LHB, Souza JTP, et al. Quality of life and sleep of teenagers with tipe 1 diabetes mellitus. Rev Bras Obes Nutr Emagrecimento. 2019 [cited 2023 Nov 17];13(81):759–65. Available from: http://www.rbone.com.br/index.php/rbone/article/view/1057.

[25] 25. Aman NF, Fitzpatrick J, de Verteuil I, Vasilevska-Ristovska J, Banh THM, Korczak DJ, et al. Family functioning and quality of life among children with nephrotic syndrome during the first pandemic wave. Pediatr Nephrol. 2023;38(9):3193–8. doi: http://dx.doi.org/10.1007/s00467-022-05809-6. PubMed PMID: 36459245. 10.1007/s00467-022-05809-6 PMC9716160 36459245

[26] 26. Fontes AP, Neri AL. Estratégias de enfrentamento como indicadores de resiliência em idosos: um estudo metodológico – ProQuest. Cien Saude Colet. 2019;24(4):1265–76. doi: http://dx.doi.org/10.1590/1413-81232018244.05502017. PubMed PMID: 31066830. 10.1590/1413-81232018244.05502017 31066830

[27] 27. Andrade RS, Santos RSFV, Santos AEV, Andrade NL, Macedo IF, Nunes MDR. Instrumentos para avaliação do padrão de sono em crianças com doenças crônicas: revisão integrativa. Rev Enf UERJ. 2018;26:31924. doi: http://dx.doi.org/10.12957/reuerj.2018.31924.

[28] 28. Rohan JM, Verma T. Psychological considerations in pediatric chronic illness: case examples. Int J Environ Res Public Health. 2020;17(5):1644. doi: http://dx.doi.org/10.3390/ijerph17051644. PubMed PMID: 32138373. 10.3390/ijerph17051644 PMC7084293 32138373

