# Supplementary material for: Children and adolescents hospitalized with chronic conditions: sleep patterns, resilience and quality of life
Source: Rev Esc Enferm USP. 2024 Apr 29;58:e20230363. doi: 10.1590/1980-220X-REEUSP-2023-0363en (PMC11089662; doi:10.1590/1980-220X-REEUSP-2023-0363en)
Supplement: Translation

# Translation: Crianças e adolescentes hospitalizados com condições crônicas: padrão de sono, resiliência e qualidade de vida*

**DOI:** 10.1590/1980-220X-REEUSP-2023-0363pt

**Authors:** Welker da Silva Xavier, Michelle Darezzo Rodrigues Nunes, Madalena Paulos Abreu, Lucila Castanheira Nascimento, Flávio Rebustini

**Affiliations:** 1 Universidade do Estado do Rio de Janeiro, Faculdade de Enfermagem, Rio de Janeiro, RJ, Brasil. Universidade do Estado do Rio de Janeiro Faculdade de Enfermagem Rio de Janeiro RJ Brasil; 2 Universidade de São Paulo, Escola de Enfermagem de Ribeirão Preto, Departamento de Enfermagem Materno-Infantil e Saúde Pública, Ribeirão Preto, SP, Brasil. Universidade de São Paulo Escola de Enfermagem de Ribeirão Preto Departamento de Enfermagem Materno-Infantil e Saúde Pública Ribeirão Preto SP Brasil; 3 Universidade de São Paulo, Escola de Artes, Ciência e Humanidades, Ribeirão Preto, SP, Brasil. Universidade de São Paulo Escola de Artes Ciência e Humanidades Ribeirão Preto SP Brasil

**Keywords:** Criança, Adolescente, Doença Crônica, Transtornos do sono-vigília, Enfermagem Pediátrica.

## Abstract

**Objetivo:**

Avaliar o padrão de sono de crianças e adolescentes com condições crônicas durante a hospitalização e correlacioná-lo com resiliência, qualidade de vida, dados clínicos e sociodemográficos.

**Método:**

Estudo quantitativo, descrito e transversal. A coleta de dados ocorreu entre maio de 2022 e janeiro de 2023, com crianças e adolescentes com condições crônicas de dois hospitais do Rio de Janeiro. Os instrumentos utilizados foram o Actigraph, Escala de Resiliência para Crianças e Adolescentes de Sandra Prince-Embury e Inventário Pediátrico de Qualidade de Vida. A análise dos dados envolveu estatística descritiva e teste de correlação.

**Resultados:**

Participaram 40 crianças e adolescentes hospitalizados entre nove e 18 anos. Os resultados mostraram sono comprometido, principalmente nos quesitos duração e tempo acordado após início do sono. Os escores para qualidade de vida foram baixos e níveis de resiliência classificados entre médio e alto. Foram encontradas correlações entre resiliência e sono. Além disso, o sono foi influenciado pelo diagnóstico e pelo tratamento.

**Conclusão:**

Crianças e adolescentes hospitalizados com condições crônicas vivenciam importantes distúrbios do sono e possuem baixa qualidade de vida, mas apresentam níveis satisfatórios de resiliência.

## INTRODUÇÃO

As crianças e os adolescentes com condição crônica precisam aprender a conviver com múltiplos, simultâneos e dinâmicos sintomas físicos, tais como dor, náusea, vômito, perda de apetite e dispneia, decorrentes do próprio diagnóstico ou de efeitos adversos e complicações do tratamento^(1)^. Como consequência, há necessidade de internação hospitalar recorrente, o que traz importantes prejuízos para o desenvolvimento social e escolar, além de sintomas psicossociais, como tristeza, preocupação e alterações na autoestima^(1)^.

Uma condição crônica pode afetar a qualidade de vida de crianças e adolescentes de inúmeras formas, mas uma das poucas discutidas é a privação do sono. Os distúrbios do sono são uma queixa comum e comprometem cerca de uma em cada quatro crianças. O reconhecimento e o manejo eficiente desses distúrbios são importantes para prevenir prejuízos no desenvolvimento físico, cognitivo, emocional, neurocomportamental e social^(2)^.

O sono configura-se como uma necessidade humana fisiológica básica e essencial para diversos âmbitos da vida e bem-estar dos seres humanos. Avaliá-lo é, portanto, um aspecto importante a ser considerado durante o exame físico global da criança e do adolescente. Quando insuficiente ou de má qualidade, inúmeras consequências podem surgir, não somente relacionadas a aspectos físicos, mas também psíquicos e sociais. Na infância, o sono torna-se ainda mais imprescindível para o crescimento e desenvolvimento físico e psicossocial^(3)^.

A prevalência de distúrbios do sono durante a infância e a adolescência é mais frequente do que se imagina, variando entre 20 e 30% da população de crianças e adolescentes^(4)^. Em crianças com condições crônicas, os principais são a piora na qualidade e na higiene do sono e a dificuldade para adormecer. Quando hospitalizadas, ainda tendem a dormir menos, devido às características do próprio diagnóstico, estrutura dos setores de internação e cuidados da equipe de saúde^(5)^.

Até mesmo após a alta hospitalar, é possível observar alterações no padrão de sono dessas crianças e adolescentes com câncer. Estudo utilizando ActiGraph em casa mostrou que tiveram duração do sono de aproximadamente seis horas e que, quanto menor esse tempo, mais propensas eram a problemas de fadiga. Constatou-se também sono interrompido por cerca de uma hora para as crianças e de duas horas para os adolescentes^(6)^.

Os efeitos de um sono fragmentado também podem ser bastante significativos na recuperação de crianças e adolescentes, pois o déficit de sono pode comprometer o sistema imune, a capacidade de resposta do organismo e a tolerância à dor^(7)^. Outras consequências incluem perda de atenção, irritabilidade, impulsividade, diminuição da tolerância a frustações, dificuldade de aprendizado, vulnerabilidade a acidentes, disfunções do humor e desenvolvimento de doenças na vida adulta, como hipertensão, diabetes e obesidade^(3)^.

Portanto, há evidências objetivas e subjetivas de distúrbios do sono frequentes em diversas condições crônicas de saúde, o que reitera a importância de considerar esse aspecto no cuidado de crianças e adolescentes. A literatura destaca, nos relatos de pior qualidade do sono durante a internação, suscetibilidade para dor e desconforto, iluminação e ruídos no local, distância da casa e ansiedade em relação aos procedimentos clínicos^(8)^. Além disso, há evidências de que esses problemas podem permanecer mesmo após a alta^(5)^.

Com isso, o objetivo geral deste estudo é avaliar o padrão de sono de crianças e adolescentes com condições crônicas durante a hospitalização. Os objetivos específicos são: explorar fatores potencialmente capazes de afetar o sono, como idade, sexo, cor de pele, diagnóstico, tratamento, tempo de internação, outras condições crônicas e motivo da internação; e correlacionar padrão de sono, resiliência e qualidade de vida em crianças e adolescentes com condições crônicas.

## MÉTODO

### Desenho do Estudo

Trata-se de um estudo quantitativo, do tipo descritivo e exploratório, de corte transversal.

### Local

Os locais do estudo foram dois hospitais de referência, na cidade do Rio de Janeiro (RJ), para o tratamento de crianças e adolescentes com condições crônicas de saúde. Foram incluídos os setores de enfermaria pediátrica, enfermaria de doenças infecciosas e enfermaria de adolescentes.

### População e Critérios de Seleção

A amostra não probabilística foi constituída por crianças e adolescentes com condições crônicas, com idades entre nove e 18 anos incompletos, internados nos dois hospitais públicos de referência selecionados para o estudo. A escolha da faixa etária se justifica, visto que os dados do sono foram correlacionados com outros que analisaram a resiliência de crianças e adolescentes com condições crônicas e a escala utilizada para essa análise se destina, exclusivamente, a essa faixa etária.

Como critérios de inclusão, foram selecionados crianças e adolescentes com diagnóstico de alguma condição crônica, com idades entre nove e 18 anos e de ambos os sexos. E, como critérios de exclusão, aqueles que se encontravam em pós-operatório imediato, internados em unidades de terapia intensiva e incapazes de responder ao questionário. Especificamente para coleta de dados referentes ao sono, incluíram-se apenas crianças e adolescentes com internação mínima nas enfermarias de três dias completos, pois, de acordo com recomendações de uso do ActiGraph, o dispositivo deve ser utilizado por, pelo menos, três dias consecutivos de 24 horas para fidedignidade dos dados^(9)^. Excluíram-se crianças e adolescentes com malformações que impossibilitassem a colocação do dispositivo ActiGraph para coleta do sono.

### Coleta de Dados

A coleta de dados ocorreu no período de maio de 2022 a janeiro de 2023. Para caracterização dos participantes, utilizou-se um formulário para síntese dos dados clínicos (diagnóstico, tempo de diagnóstico, diagnóstico de outras condições crônicas, tratamento realizado e motivo da internação) e sociodemográficos (idade, sexo, cor de pele e escolaridade).

O instrumento utilizado para coleta de dados referentes ao sono dos participantes foi o ActiGraph de pulso, que permaneceu no pulso das crianças e adolescentes por um período de, no mínimo, três dias consecutivos e completos. Os dados acumulados inicialmente foram armazenados no próprio aparelho (Mini Motionlogger ActiGraph Ambulatory Monitoring, Inc) e, após a retirada do dispositivo, transferidos, por meio de uma interface, para um notebook, através de um software específico, o WatchWare, versão 1.99.34.1. Com outro software, o Action W, versão 2.7, os dados foram processados e expressos sob a forma de actogramas. As variáveis utilizadas neste estudo foram: *sleep duration*/duração do sono, *wake after sleep onset* (WASO)/acordar após início do sono, *sleep efficiency*/eficiência do sono e *sleep percent*/porcentagem de sono. Vale ressaltar que os valores de WASO e duração do sono são calculados em minutos, enquanto a porcentagem de sono e a eficiência do sono em porcentagem^(10)^.

Actigrafia é um instrumento válido e confiável para avaliar o sono, com altas correlações entre as atividades mensuradas, tanto no punho quanto na cintura: atividade noturna, com r = 0,91 e p < 0,001; duração do sono, com r = 0,78 e p < 0,001; latência do sono, com r = 0,78 e p < 0,001; porcentagem de sono, com r = 0,89 e p < 0,001, e eficiência do sono, com r = 0,91 e p < 0,001^(11)^. Além disso, a estimativa do sono por meio do ActiGraph mostra que a correlação com a polissonografia é de cerca de 90%, considerada o padrão-ouro para detecção de distúrbios do sono^(12)^.

Para avaliar a resiliência, utilizou-se a Escala de Resiliência para Crianças e Adolescentes de Sandra Prince-Embury^(13)^, que possui versão traduzida e validada para o português, no ano de 2008, com crianças e adolescentes de nove a 18 anos^(14)^. É composta de três subescalas: controle, capacidade de relacionamento e reatividade emocional. Cada uma contém de 20 a 24 itens que, no total, avaliam dez fatores. Para cada item, há cinco alternativas de escolha, com respostas na forma *Likert*. É preenchida por meio do autorrelato da criança e do adolescente e classifica cada uma das subescalas em: baixa (≤40), abaixo da média (41 a 45), média (46 a 55), acima da média (56 a 59) e alta (≥60). Nas subescalas de controle e capacidade de relacionamento, as pontuações classificadas como na média, acima da média e alta indicam a capacidade de um indivíduo vivenciar as experiências dessas áreas de maneira satisfatória. Por outro lado, na subescala de reatividade emocional, uma pontuação acima da média ou alta indica potencial para vulnerabilidade^(13,14)^

Os parâmetros psicométricos da versão brasileira validada foram satisfatórios em relação à versão de Prince-Embury nas três escalas, indicando boa consistência interna do instrumento. O coeficiente alfa de Cronbach foi de 0,83 para a subescala controle, de 0,80 para a subescala capacidade de relacionamento, de 0,87 para a subescala reatividade emocional e de 0,93 para a escala total de resiliência^(14)^. No estudo original, a autora encontrou valores de 0,95, 0,95 e 0,94, respectivamente^(13)^.

Para avaliar qualidade de vida, utilizou-se o Inventário Pediátrico de Qualidade de Vida módulo genérico 4.0 - PedsQL™. Trata-se de um instrumento modular projetado para medir a Qualidade de Vida Relacionada à Saúde (QVRS) em crianças e adolescentes em idades de dois a 18 anos, sendo dividido por faixa etária^(15)^. Foram utilizadas as versões do autorrelato da criança (de oito a 12 anos) e do adolescente (de 13 a 18 anos). Possui quatro subescalas: funcionamento físico, funcionamento emocional, funcionamento social e funcionamento escolar. Cada escala utiliza uma escala do tipo *Likert* de cinco pontos para perguntar à criança/adolescente ou ao cuidador o quanto determinado item foi um problema ao longo da semana anterior. Os escores brutos são transformados em escalas de zero a cem, sendo 1 = 100, 2 = 75, 3 = 50, 4 = 25, 5 = 0. Escores mais altos indicam melhor QVRS^(15)^. O instrumento foi criado por Varni, Seid e Rode (1999) e validado transculturalmente em diversos países, incluindo tradução e validação para o português do Brasil, em 2008^(16)^.

Em atenção aos aspectos éticos autorais, realizou-se contato prévio com os autores das escalas para a utilização dos instrumentos, sendo a permissão concedida.

Com objetivo de assegurar a validade interna e externa do estudo para garantir resultados fidedignos e sem distorções de potenciais fontes de viés, previamente ao início da coleta de dados, a equipe de pesquisa foi treinada para padronizar a aplicação dos instrumentos de coleta de dados, reduzindo a possibilidade de erros. No que se refere à seleção da amostra, foram estabelecidos critérios claros de elegibilidade para os participantes. Além disso, durante a coleta, buscou-se garantir a representatividade da amostra, usando uma abordagem não probabilística por conveniência, a fim de garantir a inclusão de todos os estratos relevantes da população.

### Análise e Tratamento dos Dados

Para as análises estatísticas, inicialmente, foram calculados os escores obtidos das escalas de QVRS e resiliência e extraídos os dados do ActiGraph, sendo criado um banco em planilha do software Excel (Microsoft Office) para a codificação das variáveis, com dupla digitação realizada por pesquisadores distintos. Posteriormente, os dados foram exportados para o software estatístico JASP, versão 0.17.1, para realização dos testes estatísticos apropriados.

Para a caracterização da amostra, as variáveis dependentes contínuas dos dados sociodemográficas dos participantes foram descritas em média e desvio-padrão, e as variáveis dependentes nominais em distribuições de frequências absolutas (n) e relativas (%). As variáveis dependentes ordinais (escores de sono, QVRS e resiliência) foram relatadas por meio da análise das medidas de tendência central (média e mediana (Md)) e dispersão (desvio-padrão, intervalo interquartil (IIQ), mínimo, máximo e amplitude).

Visto que as respostas dos questionários na escala *Likert* são categóricas/ordinais, os valores de média e desvio-padrão foram utilizados apenas para referência, e as medidas de medianas para descrição dos resultados. Assim, as análises de variância aplicadas ao estudo foram de natureza não paramétrica.

Na análise inferencial das associações entre os escores de sono, QVRS e resiliência e características dos participantes, foram realizados testes de significância estatística entre distribuições de respostas de subgrupos distintos. Foram utilizados testes não paramétricos de Mann-Whitney, para as variáveis binárias, e Kruskal-Wallis, com post-hoc do teste de Dunn e correção de Bonferroni, para controle do erro de tipo I, para as variáveis politômicas.

Por fim, para descrever as associações entre sono, QVRS e resiliência, utilizou-se a correlação Tau de Kendall, visto o número pequeno de dados, e considerando que um coeficiente é significativamente não nulo se o valor de p do teste de correlação é < 0,05. Os valores de correlação entre 0,10 e 0,29 indicam correlação inexistente ou pequena; valores entre 0,30 e 0,49 mostram que existe correlação média e aqueles entre 0,50 e 1 podem ser interpretados como grande correlação^(17)^.

### Aspectos Éticos

Inicialmente, a proposta foi submetida ao Comitê de Ética e Pesquisa com Seres Humanos (CEP) da instituição proponente, a qual recebeu aprovação, sendo registrada sob o parecer 5.299.621. A etapa seguinte foi a obtenção do parecer de concordância da instituição coparticipante, igualmente com resposta favorável para realização da pesquisa, com parecer 5.376.441. Todo o desenvolvimento desta pesquisa foi embasado na Resolução 466/12 do Conselho Nacional de Saúde.

Cada participante do estudo, criança ou adolescente e responsável, manifestou voluntariamente seu desejo de participar ou não do estudo. Finalizou-se o processo de obtenção do consentimento com a assinatura do termo de consentimento livre e esclarecido pelos pais ou responsáveis e assinatura do termo de assentimento livre e esclarecido pelos menores participantes.

## RESULTADOS

Participaram do estudo 40 crianças e adolescentes com condições crônicas, com idades entre nove e 18 anos, hospitalizados em alguma das instituições selecionadas. Todos foram convidados a responder as escalas de resiliência e QVRS, mas apenas 26 completaram os dados referentes à avaliação do sono.

Com relação à caracterização sociodemográfica dos participantes, a idade média foi de 13,7 ± 2,6 anos. A maioria deles era adolescente com idade entre 13 e 18 anos (n = 30; 75%), e os outros 25% crianças entre nove e 12 anos (n = 10); 67,5% eram do sexo feminino (n = 27) e 32,5% do masculino (n = 13); 60% se autodeclaravam pardos (n = 24), 22,5% brancos (n = 9) e 17,5% pretos (n = 7); por fim, 77,5% cursavam o Ensino Fundamental (n = 31) e 22,5% o Ensino Médio (n = 9).

No que se refere à classificação do diagnóstico, 12 (30%) tinham alguma condição genética, 12 (30%) alguma condição autoimune e os demais (n = 16; 40%) foram agrupados como outras condições crônicas.

No momento da coleta de dados, a maioria dos participantes (n = 15; 37,5%) possuía 10 anos ou mais de tempo de diagnóstico, 22,5% entre um e três anos (n = 9), 20% entre quatro e nove anos (n = 8), 15% entre um e 12 meses (n = 6) e apenas 5% não soube responder (n = 2). A maioria não possuía o diagnóstico de outra condição crônica (n = 33; 82,5%) e apenas 17,5% apresentavam outra condição associada (n = 7). Por fim, o principal motivo de internação foi causa aguda ou clínica (n = 24; 60%), seguida de complicação da própria condição crônica (n = 8; 20%), tratamento medicamentoso (n = 6; 15%) e outras causas (n = 2; 5%). Com relação ao tratamento, 50% dos participantes realizavam a modalidade exclusivamente medicamentosa (n = 20) e os outros 50% associavam múltiplas modalidades de tratamento (n = 20).

Os dados descritivos sobre a QVRS dos participantes estão apresentados na Tabela 1. No geral, a Md dos escores de QVRS total e de suas dimensões foi baixa, indicando problemas muitas vezes ou quase sempre. A Md da QVRS total foi 60,89 (IIQ = 22,83). Ademais, de todas as dimensões, a que apresentou maior escore foi “funcionamento social” (Md = 77,50; IIQ = 26,25), e as com menor escore foram “funcionamento emocional” (Md = 50,00; IIQ = 25,00) e “funcionamento escolar” (Md = 50,00; IIQ = 31,25) (Tabela 1).

**Tabela 1:** Qualidade de vida, resiliência e variáveis do sono de crianças e adolescentes participantes da pesquisa – Rio de Janeiro, RJ, Brasil, 2023.

Subescalas	N	Md	IIQ	Amp	Mín	Máx	Média ± DP
QVRS							
Funcionamento físico	40	64,06	38,28	93,75	3,12	96,87	60,62 ± 22,86
Funcionamento emocional	40	50,00	25,00	85,00	15,00	100,00	52,00 ± 20,62
Funcionamento social	40	77,50	26,25	70,00	30,00	100,00	74,75 ± 18,50
Funcionamento escolar	40	50,00	31,25	80,00	10,00	90,00	50,05 ± 19,38
QVRS genérica total	40	60,86	22,83	59,78	25,00	84,78	59,36 ± 15,18
Resiliência							
Controle	40	52,00	20,50	54,00	22	76	50,44 ± 13,36
Capacidade de relacionamento	40	66,00	23,50	61,00	35	96	68,35 ± 15,67
Reatividade emocional	40	26,50	19,75	45,00	06	51	27,20 ± 13,54
Variáveis do sono							
WASO	26	39,62	31,65	106,05	10,20	116,25	42,47 ± 26,14
Duração do sono	26	221,27	109,35	346,03	69,22	415,25	216,42 ± 87,59
Sono, %	26	70,16	16,35	51,71	41,28	92,99	70,05 ± 14,91
Eficiência do sono	26	89,43	7,90	20,30	75,68	95,98	87,76 ± 5,89

Md: Mediana; IIQ: Intervalo Interquartil; Amp: Amplitude; Mín: Mínimo; Máx: Máximo; DP: Desvio-Padrão; QVRS: Qualidade de Vida Relacionada à Saúde; WASO: *Wake After Sleep Onset.*

Em relação à resiliência, os resultados descritivos estão apresentados na Tabela 1. As medianas dos escores das subescalas de resiliência foram 52 (IIQ = 20,50) para controle, 66 (IIQ = 23,50) para capacidade de relacionamento e 26,50 (IIQ = 19,75) para reatividade emocional. Nota-se que, com tais resultados, os participantes poderiam ser classificados com uma resiliência média para subescala controle e alta para subescala de capacidade de relacionamento, o que configurou uma capacidade satisfatória para lidar com as experiências de vida. Na subescala de reatividade emocional, a classificação da resiliência foi tida como baixa, o que não indicou vulnerabilidade para esses indivíduos (Tabela 1).

Visto que nem todas as crianças e adolescentes selecionados para pesquisa permaneceram internados por três dias completos, os dados do sono foram coletados em parte dos participantes (n = 26; 65%). Os resultados descritivos sobre o padrão de sono dos participantes estão apresentados na Tabela 1.

Notou-se que, após iniciar o sono, as crianças e os adolescentes permaneciam acordados entre 10,2 e 116,3 minutos, com Md de 39,62 minutos (IIQ = 31,65) acordados. Eles dormiam uma Md de 221,27 minutos (IIQ = 109,35), o que equivaleu, aproximadamente, a 3,7 horas, com tempo mínimo de 69,2 minutos (1,1 horas) e máximo de 415,3 minutos (6,9 horas) entre os intervalos de sono e vigília. Tal resultado evidencia o padrão de sono comprometido dos participantes do estudo.

Com relação ao percentual de sono, a Md foi de 70,16% (IIQ = 16,35), com mínimo de 41,3% e máximo de 93%. Já a eficiência do sono alcançou Md de 89,43% (IIQ = 7,90), variando entre 75,3 e 96%.

A análise inferencial das associações entre os escores de sono e características clínicas e sociodemográficas dos participantes evidenciou ausência de influência dos seguintes fatores no sono: idade, sexo, diagnóstico de outras condições crônicas, tempo de diagnóstico e motivo de internação. No entanto, a análise de variância por Kruskal-Wallis indicou violação da hipótese nula, em relação ao diagnóstico, especificamente para variável de eficiência do sono (H = 6,22; p = 0,04) (Tabela 2).

**Tabela 2:** Resultados do teste de Kruskal-Wallis para variáveis de sono de crianças e adolescentes participantes da pesquisa, segundo diagnóstico – Rio de Janeiro, RJ, Brasil, 2023.

Variáveis de sono	Fator	Estatística (H)	Df	Valor de p
WASO	Diagnóstico	3,42	2	0,18
Duração do sono	Diagnóstico	1,94	2	0,38
Sono, %	Diagnóstico	3,73	2	0,15
Eficiência do sono	Diagnóstico	6,22	2	0,04*

* p < 0,05. Df: graus de liberdade; WASO: *Wake After Sleep Onset.*

Quando aplicada a correção de Bonferroni, a violação de hipótese nula se manteve, especificamente na comparação entre condições genéticas e autoimunes (Pbonf = 0,04) (Tabela 3).

**Tabela 3:** *Post-hoc* do teste de Dunn das comparações entre diagnósticos – Rio de Janeiro RJ, Brasil, 2023.

Variáveis de sono	Comparações	Z	Wi	Wj	Pbonf
WASO	Genética – autoimune	1,75	16,43	9,50	0,24
	Genética – outras	0,51	16,43	14,54	1,00
	Autoimune – outras	–1,42	9,50	14,54	0,47
Duração do sono	Genética – autoimune	0,43	12,71	11,00	1,00
	Genética – outras	–0,84	12,71	15,82	1,00
	Autoimune – outras	–1,36	11,00	15,82	0,53
Sono, %	Genética – autoimune	0,37	11,86	10,37	1,00
	Genética – outras	–1,34	11,86	16,82	0,54
	Autoimune – outras	–1,81	10,37	16,82	0,21
Eficiência do sono	Genética – autoimune	–2,47	8,71	18,50	0,04*
	Genética – outras	–1,13	8,71	12,91	0,77
	Autoimune – outras	1,57	18,50	12,91	0,35

* p < 0,05. Wi e Wj: somas dos postos nos tratamentos i e j, respectivamente; Pbonf: p de Bonferroni; WASO: W*ake After Sleep Onset.*

Nas análises descritivas, nota-se que os participantes com diagnóstico de condições genéticas (Md = 83,55; IIQ = 2,26) possuíam eficiência de sono inferior, quando comparados àqueles com condições autoimunes (Md = 91,90; IIQ = 3,61) (Tabela 4).

**Tabela 4:** Variáveis de sono de crianças e adolescentes participantes da pesquisa, segundo diagnóstico e tratamento – Rio de Janeiro, RJ, Brasil, 2023.

Variáveis de sono	Grupos	N	Md	IIQ	Amp	Mín	Máx	Média ± DP
WASO	Genética	7	44,60	29,47	79,33	21,50	100,83	51,91 ± 27,31
	Autoimune	8	28,66	17,18	60,80	10,20	71,00	29,52 ± 19,33
	Outras	11	46,00	28,04	104,12	12,13	116,25	45,87 ± 28,10
Duração do sono	Genética	7	199,57	82,36	207,07	88,50	295,57	200,64 ± 71,83
	Autoimune	8	185,73	105,47	293,89	84,44	378,33	195,37 ± 96,54
	Outras	11	246,44	68,72	346,03	69,22	415,25	241,78 ± 91,25
Sono, %	Genética	7	70,01	15,21	41,02	41,28	82,30	66,54 ± 14,89
	Autoimune	8	63,92	17,97	48,30	42,70	91,00	64,46 ± 16,53
	Outras	11	78,10	14,15	42,98	50,01	92,99	76,35 ± 12,42
Eficiência do sono	Genética	7	83,55	2,26	18,81	75,68	94,49	84,22 ± 5,57
	Autoimune	8	91,90	3,61	20,06	75,92	95,98	90,92 ± 6,42
	Outras	11	88,84	4,06	15,02	78,10	93,12	87,71 ± 4,76
WASO	Medicamentoso	15	46,00	40,67	105,00	11,25	116,25	50,72 ± 29,96
	Múltiplo	11	30,33	17,12	44,940	10,20	55,14	31,21 ± 14,48
Duração do sono	Medicamentoso	15	199,57	106,41	330,81	84,44	415,25	216,23 ± 89,14
	Múltiplo	11	230,71	96,49	309,11	69,22	378,3	216,69 ± 89,76
Sono, %	Medicamentoso	15	70,31	15,38	51,60	41,28	92,88	70,08 ± 15,09
	Múltiplo	11	70,02	15,65	50,23	42,76	92,99	70,01 ± 15,40
Eficiência do sono	Medicamentoso	15	84,37	7,41	20,21	75,68	95,89	85,02 ± 6,29
	Múltiplo	11	91,00	1,95	8,08	87,90	95,98	91,50 ± 2,17

Md: Mediana; IIQ: Intervalo Interquartil; Amp: Amplitude; Mín: Mínimo; Máx: Máximo; DP: Desvio-Padrão; WASO: *Wake After Sleep Onset.*

Além disso, quando o sono foi avaliado segundo o tratamento realizado, os resultados do teste de Mann-Whitney também indicaram violação da hipótese nula, especificamente para variável de eficiência do sono (W = 31,00; p = 0,01) (Tabela 5).

**Tabela 5:** Resultados do teste de Mann-Whitney para variáveis de sono de crianças e adolescentes participantes da pesquisa, segundo tratamento – Rio de Janeiro, RJ, Brasil, 2023.

Variáveis de sono	Estatística (W)	*Rank-biserial*	VS-MPR	Valor de p
WASO	116,00	0,41	1,73	0,09
Duração do sono	80,00	–0,03	1,00	0,92
Sono, %	85,00	0,03	1,00	0,92
Eficiência do sono	31,00	–0,62	11,31	0,01*

* p < 0,05. VS-MPR: *Vovk-Sellke maximum p-ratio*; WASO: *Wake After Sleep Onset.*

Observa-se que as crianças e adolescentes com tratamento exclusivamente medicamentoso possuíam uma eficiência do sono pior (Md = 84,37; IIQ = 7,41) quando comparados àqueles que acrescentaram outras modalidades de tratamento (Md = 91,00; IIQ = 1,95) (Tabela 4).

Em relação aos testes de correlações, não foram encontradas correlações significativas entre o escore total de QVRS, bem como suas dimensões de funcionamento físico, emocional, social e escolar, com nenhuma das quatro variáveis de sono utilizadas (acordar após início do sono, duração do sono, porcentagem de sono e eficiência do sono).

Encontrou-se média correlação positiva entre a subescala de reatividade emocional de resiliência e a variável acordar após início do sono (r = 0,39; p = 0,005), além de média correlação negativa com a eficiência do sono (r = –0,31; p = 0,03), ou seja, participantes com menor escore de reatividade emocional e, consequentemente, melhor resiliência, acordaram menos após o início do sono e tiveram melhor eficiência do sono. Não foram encontradas correlações entre as demais subescalas de resiliência e outras variáveis de sono.

## DISCUSSÃO

Segundo a Sociedade Portuguesa do Sono, para crianças e adolescentes com idades entre seis e 13 anos, recomenda-se tempo de sono entre nove e 11 horas; entre 14 e 17 anos de idade, cerca de oito a 10 horas; e, a partir dos 18 anos de idade, entre sete e nove horas^(18)^. Logo, percebe-se que os indivíduos participantes do estudo possuíam tempo total de sono muito abaixo do recomendado. Além disso, um sono adequado e de qualidade é definido por: uma eficiência de, pelo menos, 85%; conseguir adormecer em 30 minutos ou menos; despertar no máximo uma vez durante a noite; e permanecer acordado por menos de 20 minutos depois do início do sono^(19)^. Com isso, também pode-se observar comprometimento do tempo que as crianças do estudo permaneciam acordadas após a fase inicial do sono.

Corroborando os achados acima, novo estudo multicêntrico, com Brasil, Portugal e Estados Unidos, com o uso do ActiGraph, mostrou que, durante a internação, crianças e adolescentes com câncer têm os valores de percentual e eficiência de sono diminuídos (aproximadamente 59% e 79%, respectivamente), independentemente do sintoma de dor^(20)^. Existem inúmeros riscos relativos à privação do sono, como a presença de distúrbios físicos e psicológicos de curto e longo prazo. Quando o tempo total de sono ainda é inferior a quatro horas, a diminuição do desempenho cognitivo dos indivíduos é potencializada^(3)^.

No estudo, não foram encontradas violações de hipótese nula para as variáveis de avaliação do sono, segundo idade e sexo. Resultados semelhantes foram encontrados em estudo com crianças e adolescentes entre 10 e 18 anos com diabetes mellitus tipo I, para as quais a idade e o sexo não foram preditores para uma maior chance de sonolência diurna^(21)^. Também existem resultados compatíveis com crianças e adolescentes com câncer, e a idade também não foi um preditor para menor duração do sono^(6)^.

Para os demais dados sociodemográficos, como local do estudo, cor de pele, escolaridade, sexo escolaridade e estado civil do responsável, procedência e renda familiar, não foram encontradas violações de hipóteses nulas para as variáveis de sono. No entanto, outra pesquisa corrobora esse resultado. Em trabalho realizado com crianças e adolescentes entre 13 e 18 anos com gastrite crônica, constatou-se que, no grupo com renda menor do que os reais gastos financeiros, a qualidade do sono foi significativamente pior^(22).^

Foi encontrada, no presente estudo, violação de hipótese nula para eficiência de sono segundo o diagnóstico dos participantes (Pbonf = 0,04), mostrando que aqueles com condições crônicas genéticas possuíam menor eficiência, quando comparados àqueles com condições autoimunes. O tratamento também influenciou negativamente a variável de eficiência do sono, e o grupo de participantes em tratamento exclusivamente medicamentoso possuía valores menores, quando comparado ao de múltiplo tratamento.

Em acordo com esses resultados, em estudo com 30 crianças com fibrose cística, a análise de polissonografia mostrou redução da eficiência do sono (66,5%), índice de despertar de 8,7%, índice de distúrbio respiratório com Md de 2,6% e síndrome da apneia obstrutiva do sono em 30% dos participantes^(23)^.

Tempo de diagnóstico, motivo de internação e diagnóstico de outras condições crônicas também não interferiram significativamente nas variáveis de sono, porém, em outros estudos, foram encontrados resultados opostos. Em crianças e adolescentes com diabetes mellitus tipo I, existe maior prevalência de sonolência diurna entre os pacientes com menos de 3 anos de diagnóstico, quando comparados com aqueles com 3 anos ou mais (57,1% versus 36,2%; p = 0,03). No entanto, o tratamento com insulina não teve influência na sonolência diurna (p = 0,80)^(21)^.

Não foram encontradas correlações significativas entre o escore total de QVRS e suas dimensões com nenhuma das quatro variáveis de sono utilizadas. Em contrapartida, outros estudos encontraram associações, mostrando a influência entre QVRS e sono. Estudo realizado com adolescentes entre 10 e 16 anos, com diabetes mellitus tipo I, mostrou correlação significativa (p = 0,02) entre qualidade do sono, medida pelo PSQI, e domínio físico do questionário *World Health Organization Quality of Life bref* (WHOQOL-bref). Nesses indivíduos, quanto pior a qualidade do sono, pior a qualidade de vida no domínio físico^(24).^

Estudos mais recentes também mostraram influência da pandemia da Covid-19 sobre a qualidade de vida e o sono de crianças e adolescentes com condições crônicas. Em sujeitos com síndrome nefrótica, entre 6 meses e 18 anos, a QVRS geral diminuiu (p = 0,04), ao mesmo tempo em que aumentou a interrupção do sono e diminuiu a energia (p = 0,01) durante a pandemia. Além disso, o aumento da idade da criança também foi preditor para maior interrupção do sono e diminuição na qualidade de vida geral^(25)^.

Com relação à resiliência e ao sono, identificou-se, no presente estudo, que quanto maior o escore da reatividade emocional de resiliência, maior o tempo de permanência acordado após iniciar o sono, e menor a eficiência do sono. Estratégias de enfrentamento também são importantes indicadores de resiliência e desempenho da reatividade emocional, que consiste na capacidade de manter o equilíbrio após perturbações ou fortes excitações. Essas estratégias representam modificações comportamentais e cognitivas, por meio das quais os indivíduos alteram o ambiente e as emoções para lidar com situações desafiadoras e estressantes^(26)^.

Quando a condição crônica surge, essas estratégias de enfrentamento positivas também são necessárias para restabelecer o equilíbrio entre as complicações físicas, sinais e sintomas e satisfação de suas necessidades, bem como as limitações sociais, como evasão escolar. Como repercussão desse isolamento e limitações, diversos sintomas podem ser desencadeados ou exacerbados, incluindo ansiedade, depressão, insônia e sonolência excessiva^(1)^.

Ainda que comprovada a relevância da temática, é reconhecida a carência de pesquisas na população de crianças e adolescentes com condições crônicas, o que evidencia a importância do presente estudo. Contudo, foram encontradas algumas limitações, que constituem possibilidades para novas pesquisas. Sabe-se que o tamanho da amostra é pequeno, fato justificado pela necessidade de uma internação prolongada dos participantes selecionados para pesquisa, e isso pode interferir significativamente nos seus resultados.

Estudos semelhantes a este^(6,11,20)^ constituem-se em desafios aos pesquisadores, dada a escassez de literatura relacionando o sono e as variáveis aqui utilizadas. Isso reitera a fragilidade e a lacuna de estudos na área, sendo um importante tópico a ser abordado em estudos futuros, que ultrapassem as limitações encontradas.

Sabe-se que crianças e adolescentes com condições crônicas são mais suscetíveis a desenvolverem distúrbios do sono, como diminuição da qualidade e da higiene do sono, despertares noturnos e dificuldade para iniciar o sono. Em situações de internação, esses problemas são exacerbados, pelas características da própria condição, tratamento, múltiplos sintomas, ruídos e iluminação e cuidados da equipe de enfermagem^(27)^. Nesse contexto, diversas estratégias podem ser utilizadas para prestar uma assistência de enfermagem segura, de qualidade e holística, como uso de brinquedo terapêutico, técnicas de distração durante procedimentos, imaginação guiada, relaxamento com respiração profunda e realização de desenhos, por exemplo^(28)^.

## CONCLUSÃO

Atendendo ao objetivo da pesquisa, os resultados mostram importante comprometimento do sono dessa população durante o período de internação. Além disso, identificou-se pior eficiência do sono em participantes com diagnóstico de condições genéticas e em tratamento medicamentoso exclusivo. Em relação à resiliência, quanto melhor a reatividade emocional, menor o tempo de permanência dos participantes acordados após a fase inicial do sono e melhor a eficiência do sono.

Sabe-se que consequências da privação de sono também precisam ser avaliadas pelos profissionais da saúde, visto que essa privação torna aos indivíduos mais suscetíveis para um atraso no crescimento e desenvolvimento, desenvolvimento de outras condições crônicas, dificuldade de aprendizado, alterações de comportamento e prejuízo cognitivo.

Assim, a primeira implicação para a enfermagem refere-se à inclusão da avaliação do padrão de sono, por meio de instrumentos válidos e confiáveis no planejamento de suas atividades. Para tanto, o enfermeiro precisa ter conhecimento da estrutura e da duração do sono para cada faixa etária, além dos instrumentos disponíveis para este fim.

Evidenciada a presença de distúrbios do sono em crianças e adolescentes com condições crônicas durante a hospitalização, torna-se urgente a implementação de estratégias para minimizá-los. Isso pode ser feito, por exemplo, por meio da manutenção de um ambiente aconchegante e de práticas de massagem e musicoterapia, que podem ser incluídas no processo de enfermagem, para garantia de um sono de qualidade nessa população.

Logo, ressalta-se a importância de avaliar precocemente o padrão de sono, para que crianças e adolescentes possam ter melhor qualidade de vida e resiliência durante o período de hospitalização e tratamento, por meio do gerenciamento adequado desse sintoma. No entanto, novos estudos, com maior número de participantes e tempo de coleta de dados mais prolongado, são necessários e devem ser estimulados, para aprofundamento desta temática na faixa etária infantojuvenil.
